# Inhibitory effect of *Citrus* flavonoids on the in vitro transport activity of human urate transporter 1 (URAT1/SLC22A12), a renal re-absorber of urate

**DOI:** 10.1038/s41538-020-0063-7

**Published:** 2020-02-05

**Authors:** Yu Toyoda, Tappei Takada, Hiroki Saito, Hiroshi Hirata, Ami Ota-Kontani, Naoyuki Kobayashi, Youichi Tsuchiya, Hiroshi Suzuki

**Affiliations:** 1 Department of Pharmacy, The University of Tokyo Hospital, 7-3-1 Hongo, Bunkyo-ku, Tokyo, 113-8655 Japan; 2 Frontier Laboratories for Value Creation, SAPPORO HOLDINGS LTD., 10 Okatome, Yaizu, Shizuoka, 425-0013 Japan

**Keywords:** Biochemistry, Nutrition

## Abstract

As hyperuricemia is a cause of urate-related diseases such as gout, the anti-hyperuricemic and/or uricosuric activity of food ingredients is receiving increased attention. Here, we examined the inhibitory activities of seven *Citrus* flavonoids against URAT1, a renal transporter involved in urate re-uptake from urine. We found that naringenin and nobiletin strongly inhibited URAT1, and may therefore serve as an anti-hyperuricemic food ingredient that can reduce the risk of urate-related diseases.

## Introduction

Hyperuricemia, a condition characterized by high level of serum uric acid (SUA), often originates from excessive production and/or decreased renal/intestinal excretion of uric acid.^1^ Recently, hyperuricemia and related pathological conditions such as gout have become major public health issues with highly prevalence.^1^ SUA management at appropriate levels via the daily consumption of foods that can reduce hyperuricemia risk is therefore becoming increasingly important.

We previously determined that allopurinol, the oldest xanthine oxidase (XO, a key enzyme to catalyze uric acid production) inhibitor, moderately inhibited urate transporter 1 (URAT1, a physiologically important renal urate transporter).^2^ Together with the fact that XO and urate transporters interact with urate, these findings led to the suggestion that some XO inhibitors can inhibit URAT1. A dual XO and URAT1 inhibitor has been synthesized, although its development was terminated due to safety risks.^3^ If we consumed nutrients with such inhibitory activity in daily life, this may contribute to health maintenance. We therefore designed the present study to seek URAT1 inhibitors from natural food ingredients exhibiting XO-inhibitory activity. Interestingly, a previous study demonstrated that seven flavonoids isolated from *Citrus* fruits (Supplementary Fig. 1) inhibited XO.^4^ Thus, we evaluated their URAT1-inhibitory activities using a cell-based assay.

## Results

**Fig. 1:**
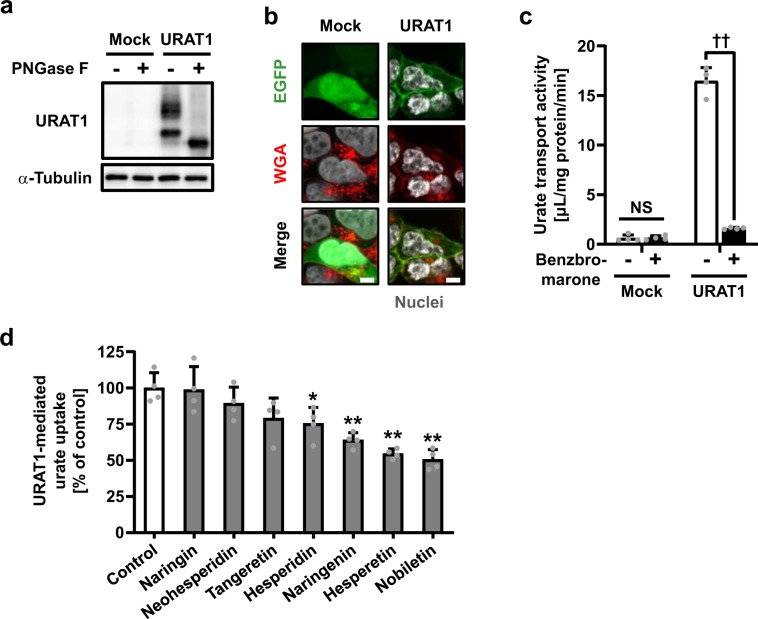
Effect of seven *Citrus* flavonoids on URAT1-mediated urate transport. **a**, **b** Expression and plasma membrane localization of URAT1 in 293A cells. A fluorescent wheat germ agglutinin conjugate (WGA) was used to visualize plasma membranes. Bars, 5 μm. **c** URAT1 inhibition by benzbromarone (10 μM). ^††^*P* < 0.01 (two-sided *t*-test); NS not significant. **d** Inhibitory effect of each flavonoid (15 μM) on URAT1-mediated urate transport. Data are expressed as the mean ± SD. *n* = 4. *P < 0.05; **P < 0.01 vs control (Dunnett’s test).

Immunoblotting and confocal microscopic observation confirmed the expression and plasma membrane localization of EGFP-tagged URAT1 (EGFP-URAT1) in cells transiently expressing URAT1 (Fig. 1a, b). Benzbromarone,^5^ a URAT1 inhibitor, inhibited 96% of URAT1-mediated urate uptake into the cells (Fig. 1c). This result is consistent with a previous report using non-tagged URAT1.^6^

**Fig. 2:**
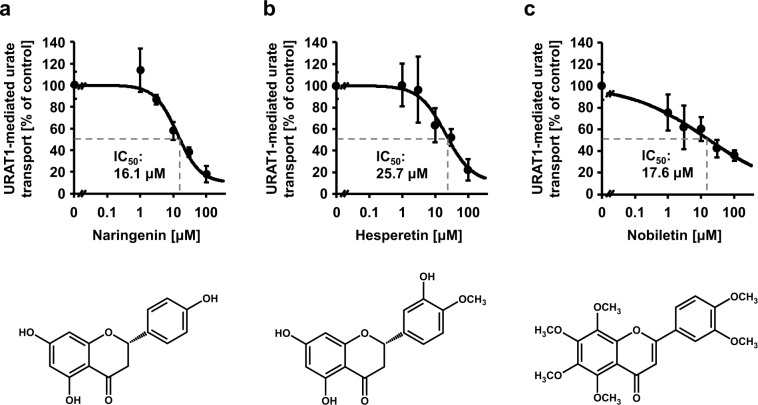
Concentration-dependent inhibition of URAT1-mediated urate transport by naringenin, hesperetin, and nobiletin. Data are expressed as the mean ± SD. *n* = 4.

After verifying the assay system, we examined the inhibitory effect of seven *Citrus* flavonoids (15 μM) on URAT1 function (Fig. 1d). Among them, one polymethoxyflavonoid (nobiletin) and two flavanones (hesperetin and naringenin) inhibited URAT1 (greater than 30%) more strongly than the others. Whereas, two flavanone glycosides (naringin and neohesperidin) exhibited little inhibitory effect, as the presence of glycone had disrupted the XO-inhibitory activities of the flavanones.^4^ We further examined the concentration-dependent inhibitory effects of naringenin, hesperetin, and nobiletin on URAT1 (Fig. 2). Despite the comparable values of the half maximal inhibitory concentration (IC_50_) against URAT1, nobiletin inhibited URAT1 activity more strongly than naringenin at low concentrations (≤3 μM). Additionally, a cell-based cytotoxic assay showed that the three flavonoids, under or around the calculated IC_50_ concentrations (Fig. 2), had little effect on cytotoxicity (Supplementary Fig. 2).

## Discussion

Here, we newly identified some URAT1-inhibitors from *Citrus* flavonoids. Among them, naringenin, hesperetin, and nobiletin showed substantial URAT1 inhibitory activities; the IC_50_ values were 16.1 μM, 25.7 μM, and 17.6 μM, respectively. A previous study determined the XO inhibitory activities of these three flavonoids, with IC_50_ values of >200 μM (naringenin), 16.5 μM (hesperetin), and 107.5 μM (nobiletin).^4^ Considering the one-digit differences between the values under the assumption of uniformity of each flavonoid level through the body due to the limitation in available information, naringenin, and nobiletin appear to affect URAT1 more than XO at lower concentrations. Although further investigations in humans based on this study are highly warranted, our findings can extend studies of applying flavonoids to reduce hyperuricemia risk as exampled by the case in morin—a natural fravonol.^7^ With the three flavonoids, some in vivo studies implied their potential as anti-hyperuricemic food ingredients as follows.

First, oral administration of the aglycon of naringenin (100 mg/kg) for 3 days reportedly lowered SUA in hyperuricemic mice.^8^ However, the mechanisms underlying this hypouricemic action remain unclear; no information was available on the blood concentration of naringenin in the mice, unfortunately. With this matter, our results provide a plausible explanation that the SUA-lowering phenotype might be due to naringenin-mediated URAT1 inhibition rather than XO inhibition.

Second, a hypouricemic effect was observed in hyperuricemia model rats orally supplemented with orange juice (5 mL/kg/day) for 2 weeks.^9^ The dominant flavonoids in oranges are hesperidin and narirutin that are hydrolyzed to hesperetin and naringenin (their corresponding aglycons, respectively) in the colon and then absorbed into the body. Thus, the beneficial effects of orange juice may be due to hesperetin and/or naringenin, though the associated mechanisms remain to be elucidated. As fruit juices are good sources of *Citrus* flavonoids,^10^ evaluating the effect of long-term *Citrus* fruit and juice consumption on SUA in humans would be beneficial. Interestingly, regular consumption of orange fermented beverage (500 mL/day) every day for 2 weeks reportedly reduced SUA, significantly (−8.9%), in healthy subjects.^11^

Third, *Citrus depressa* extracts containing nobiletin exhibited uricosuric effects in hyperuricemia model mice,^12^ and nobiletin was present in the urine of nobiletin-fed mice.^13^ Given this information, nobiletin could suppress renal urate re-absorption, resulting in the improvement of hyperuricemia. Our findings suggest that nobiletin-mediated URAT1 inhibition would be involved in this biological response. Considering the presence of some types of demethylated nobiletins in the urine of nobiletin-fed mice,^13^ the URAT1-inhibitory activity of such metabolites will become an issue in the future. Moreover, there is little information about the kinetics of nobiletin, especially in humans. Therefore, both in vivo and human studies need to be conducted to extend our findings.

In conclusion, we discovered three candidates for functional food ingredient URAT1 inhibitors from *Citrus* flavonoids. In spite of the similar chemical structures of naringenin, hesperetin, and nobiletin, their urate modification properties are distinguishable. Clarifying their potential for serving as anti-hyperuricemic food ingredients will provide deeper insight into health benefit of dietary flavonoids.

## Methods

### Materials

Critical materials and resources used in this study are summarized in Supplementary Table 1. All other chemicals used were commercially available and of analytical grade.

### Cell culture

Human embryonic kidney 293 (HEK293)-derived 293A cells (Invitrogen, Carlsbad, CA, USA) were maintained in Dulbecco’s Modified Eagle’s Medium (Nacalai Tesque, Kyoto, Japan) supplemented with 10% fetal bovine serum (Biowest, Nuaillé, France), 1% penicillin-streptomycin (Nacalai Tesque), 2 mM l-Glutamine (Nacalai Tesque), and 1x Non-Essential Amino Acid (Life Technologies, Tokyo, Japan) at 37 °C in a humidified atmosphere of 5% (v/v) CO_2_ in air.

Each vector plasmid for URAT1 (URAT1 wild type in pEGFP-C1) or mock (pEGFP-C1) was transfected into 293A cells by using polyethyleneimine “MAX” (PEI-MAX) (Polysciences, Warrington, PA, USA) as described previously,^14^ with some modifications. In brief, before transfection, 293A cells were seeded onto cell culture 12-well plates at a concentration of 0.92 × 10^5^ cells/cm^2^. Twenty-four hours after seeding, each plasmid vector was transiently transfected into the cells using PEI-MAX (1 μg of plasmid/5 μL of PEI-MAX/well). The medium was replaced with fresh medium after the first 24 h of incubation.

### WST-8 assay

To evaluate the effect of flavonoids on cytotoxicity, the viabilities of the 293A cells cultured in the presence of each flavonoid were detected using a Cell Count Reagent SF (Nacalai Tesque) as described previously^15^ with some modifications. In brief, cells were seeded at 5 × 10^4^ cells/well into 96-well cell culture plates and pre-cultured for 24 h. After incubating for 48 h with each flavonoid at various concentrations, the culture medium was replaced with 100 μL fresh medium containing 10% (v/v) of the reagent for the WST-8 assay. After 1 h of further incubation, the absorbance at 450 nm derived from WST-8-formazan in culture medium was measured using a Varioskan^TM^ Flash Multimode Reader (Thermo Fisher Scientific, Yokohama, Japan).

### Preparation of protein lysates

Whole-cell lysates were prepared with cell lysis buffer A containing 50 mM Tris/HCl (pH 7.4), 1 mM dithiothreitol, 1% (w/v) Triton X-100, and a protease inhibitor cOmplete, EDTA free (Roche, Basel, Switzerland). They were then treated with Peptide *N*-glycosidase F (PNGase F) (New England Biolabs, Ipswich, MA, USA) as described previously.^15^ The protein concentration was determined by using the Pierce^TM^ BCA Protein Assay Kit (Thermo Fisher Scientific) with BSA as a standard according to the manufacturer’s protocol.

### Immunoblotting

Whole-cell lysate samples were separated by SDS-PAGE and transferred to an Immobilon-P PVDF membrane (Millipore, Bedford, MA, USA) by electroblotting at 15 V for 60 min. For blocking, the membrane was incubated in Tris-buffered saline containing 0.05% Tween 20 and 3% BSA (TBST-3% BSA). Blots were probed with appropriate antibodies (Supplementary Table 1), and the signals were visualized by chemiluminescence. All antibodies were used at a 1:1000 (first antibodies—Rabbit polyclonal anti-EGFP; Cat# A11122; Life Technologies: Rabbit polyclonal anti-α-tubulin; Cat# ab15246; Abcam, Cambridge, MA, USA) or 1:2,000 (second antibody—Donkey anti-rabbit IgG-horseradish peroxidase (HRP)-conjugate; Cat# NA934V; GE Healthcare, Buckinghamshire, UK) dilution in TBST-0.1% BSA for 1 h at room temperature. After washing by TBST for 1 h at room temperature, HRP-dependent luminescence was developed with ECL^TM^ Prime Western Blotting Detection Reagent (GE Healthcare) and detected using a multi-imaging Analyzer Fusion Solo 4^TM^ system (Vilber Lourmat, Eberhardzell, Germany). Uncropped scans of the blots were shown in Supplementary Fig. 3.

### Confocal microscopic observation

For confocal laser scanning microscopic observation, 48 h after the transfection, 293A cells were fixed with 4% paraformaldehyde for 15 min at room temperature, and washed three times with PBS (−). To visualize plasma membranes, the cells were then treated with a fluorescent wheat germ agglutinin conjugate (WGA, Alexa Fluor® 594 conjugate; Thermo Fisher Scientific) (10 μg/mL) in PBS (−) for 10 min at room temperature. After washing with PBS (−), the cells were treated with PBS (−) containing 0.02% (w/v) Triton X-100 for 5 min, then subjected to TO-PRO-3 Iodide (Molecular Probes, Eugene, OR, USA) staining for 10 min at room temperature in the dark. After visualizing the nuclei, the cells were washed by PBS (−) twice and mounted in VECTASHIELD Mounting Medium (Vector Laboratories, Burlingame, CA, USA). To analyze the localization of EGFP-fused URAT1 protein, fluorescence was observed using the FV10i Confocal Laser Scanning Microscope (Olympus, Tokyo, Japan).

### Urate uptake assay using URAT1-expressing 293A cells

The [8-^14^C]-urate transport activity of URAT1, in the presence or absence of test compounds at the indicated concentrations with 0.1% dimethyl sulfoxide (vehicle control), was evaluated using URAT1-expressing 293A cells according to our previous study,^2^ with minor modifications. In brief, 48 h after plasmid transfection, the cells were washed twice with Cl^−^-free transport buffer (Buffer T2: 125 mM Na-gluconate, 4.8 mM K-gluconate, 1.2 mM KH_2_PO_4_, 1.2 mM MgSO_4_, 1.3 mM Ca-gluconate, 25 mM HEPES, 5.6 mM d-glucose, and pH 7.4) and pre-incubated in Buffer T2 for 15 min at 37 °C. The buffer was then exchanged with pre-warmed fresh Buffer T2 containing 5 μM [8-^14^C]-urate with or without test compound at the indicated concentrations. The cells were further incubated for 20 s. The cells were subsequently washed twice with ice-cold Buffer T2 and then lysed with 500 μL 0.2 M NaOH on ice with gentle shaking for 1 h. The resulting lysates were neutralized with 100 μL 1 M HCl. We then measured the radioactivity in the lysate using a liquid scintillator (Tri-Carb 3110TR, PerkinElmer, Waltham, MA, USA). The protein concentrations were determined using the Pierce^TM^ BCA Protein Assay Kit. The urate transport activity was calculated as the incorporated clearance (μL/mg protein/min): (incorporated level of urate [DPM/mg protein/min]/urate level in the incubation mixture [DPM/μL]). URAT1-dependent urate transport activity was calculated by subtracting the urate transport activity of mock cells from that of the URAT1-expressing cells.

To address IC_50_ values of each test compound against the urate transport by URAT1, the urate transport activities were measured in the presence of test compounds at several concentrations. The URAT1-mediated transport activities were then expressed as a percentage of control (100%). Based on the calculated values, fitting curves were obtained according to the following formula (1) using the least-squares methods with the Excel 2013 (Microsoft, Redmond, WA, USA):

1 $${\mathrm{Predicted}}\,{\mathrm{value}}\,\left[ {\mathrm{\% }} \right] = 100 - \left( {E_{{\mathrm{max}}} \times C^n/{\mathrm{EC}}_{50}^n + C^n} \right)$$

where, *E*_max_ is the maximum effect, EC_50_ is the half maximal effective concentration, *C* is the concentration of the test compound, and *n* is the sigmoid-fit factor. Finally, based on the results, the IC_50_ was calculated.

### Quantification and statistical analysis

Unless otherwise noted, data are expressed as mean ± SD, *n* (the number of biological replicates) = 4. All statistical analyses were performed using Excel 2013 (Microsoft) with Statcel3 add-in software (OMS publishing, Saitama, Japan). Different statistical tests were used for different experiments as described in the figure legends. Briefly, when analyzing multiple groups, the similarity of variance between groups was compared using Bartlett’s test. When passing the test for homogeneity of variance, a parametric Dunnett’s test was used. In the case of a single pair of quantitative data, after comparing the variances of a set of data by an *F*-test, an unpaired Student’s *t*-test was performed. Statistical significance was defined in terms of *P* < 0.05 or 0.01.

Each experiment was designed to use the minimum number of samples required to obtain informative results and sufficient material for subsequent studies. No specific statistical test was used to pre-determine the sample sizes empirically determined in the current study. All experiments were monitored in a non-blinded fashion. Samples that had undergone technical failure during processing were excluded from analyses.

## Supplementary information

Supplementary Information

## Data Availability

Data supporting the findings of this study are included in this published article and its Supplementary Information or the datasets generated and/or analyzed during the current study are available from the corresponding author on reasonable request.
