# Supplementary material for: Inhibitory effect of Citrus flavonoids on the in vitro transport activity of human urate transporter 1 (URAT1/SLC22A12), a renal re-absorber of urate
Source: NPJ Sci Food. 2020 Feb 5;4:3. doi: 10.1038/s41538-020-0063-7 (PMC7002704; doi:10.1038/s41538-020-0063-7)
Supplement: Supplementary file 1 — Supplementary Information [file 41538_2020_63_MOESM1_ESM.pdf]

SUPPLEMENTARY INFORMATION

Inhibitory effect of *Citrus* flavonoids on the *in vitro* transport activity of human urate transporter 1 (URAT1/SLC22A12), a renal re-absorber of urate

Yu Toyoda[1,*], Tappei Takada[1,*], Hiroki Saito[1,2,*], Hiroshi Hirata[2], Ami Ota-Kontani[2], Naoyuki Kobayashi[2], Youichi Tsuchiya[2], and Hiroshi Suzuki[1]

[1] Department of Pharmacy, The University of Tokyo Hospital, 7-3-1 Hongo, Bunkyo-ku, Tokyo 113-8655, Japan

[2] Frontier Laboratories for Value Creation, SAPPORO HOLDINGS LTD., 10 Okatome, Yaizu, Shizuoka 425-0013, Japan

[*] These authors contributed equally to this work

Correspondence: tappei-tky@umin.ac.jp (TT)

Supplementary Table 1

Supplementary Figures 1–3

Supplementary References

Supplementary Table 1 Key resources.

REAGENT or RESOURCE	SOURCE	IDENTIFIER
Antibodies		
Rabbit polyclonal anti-EGFP	Life Technologies	Cat# A11122; RRID: AB_221569
Rabbit polyclonal anti-α-tubulin	Abcam	Cat# ab15246; RRID: AB_301787
Donkey anti-rabbit IgG-horseradish peroxidase (HRP)-conjugate	GE Healthcare	Cat# NA934V; RRID: AB_772206
Chemicals		
[8-^{14}C]-uric acid (53 mCi/mmol)	American Radiolabeled Chemicals	Cat# ARC0513
Benzbromarone	FUJIFILM Wako Pure Chemical Corporation	Cat# 028-1585; CAS: 3562-84-3; Purity: >98%
Clear-sol II	Nacalai Tesque	Cat# 09136-83
Dimethyl Sulfoxide	Nacalai Tesque	Cat# 13445-74; CAS: 67-68-5
Hesperetin	FUJIFILM Wako Pure Chemical Corporation	Cat# 320-93841; CAS: 520-33-2; Purity: >96%
Hesperidin	FUJIFILM Wako Pure Chemical Corporation	Cat# 088-07341; CAS: 520-26-3; Purity: >95%
Naringenin	Combi-Blocks	Cat# QA-3717; CAS: 480-41-1; Purity: >98%
Naringin	ChromaDex	Cat# ASB-00014222-001; CAS: 10236-47-2; Purity: not available
Neohesperidin	Combi-Blocks	Cat# QV-2102; CAS: 13241-33-3; Purity: >98%
Nobiletin	FUJIFILM Wako Pure Chemical Corporation	Cat# 149-09341; CAS: 478-01-3; Purity: >98%
Polyethelenimine “MAX” (PEI-MAX)	Polysciences	Cat# 24765; CAS: 49553-93-7
Tangeretin	FUJIFILM Wako Pure Chemical Corporation	Cat# 208-15671; CAS: 481-53-8; Purity: >95%
Critical Commercial Assays		
Pierce™ BCA Protein Assay Reagent A	Thermo Fisher Scientific	Cat# 23223
Pierce™ BCA Protein Assay Reagent B	Thermo Fisher Scientific	Cat# 23224
PureLink™ HiPure Plasmid Filter Midiprep Kit	Thermo Fisher Scientific	Cat# K210015
Recombinant DNA		
The complete human URAT1 cDNA	Miyata et al, 2016[1]	NCBI Reference Sequence: NM_144585.3
Experimental Models: Cell Lines		
293A	Invitrogen	R70507
Software and Algorithms		
Excel 2013	Microsoft	https://products.office.com/ja-jp/microsoft-excel-2013
Statcel3 add-in software	OMS Publishing	http://www.oms-publ.co.jp/

Information on purity was from data sheets of each chemical provided by suppliers.

Supplementary Figure 1

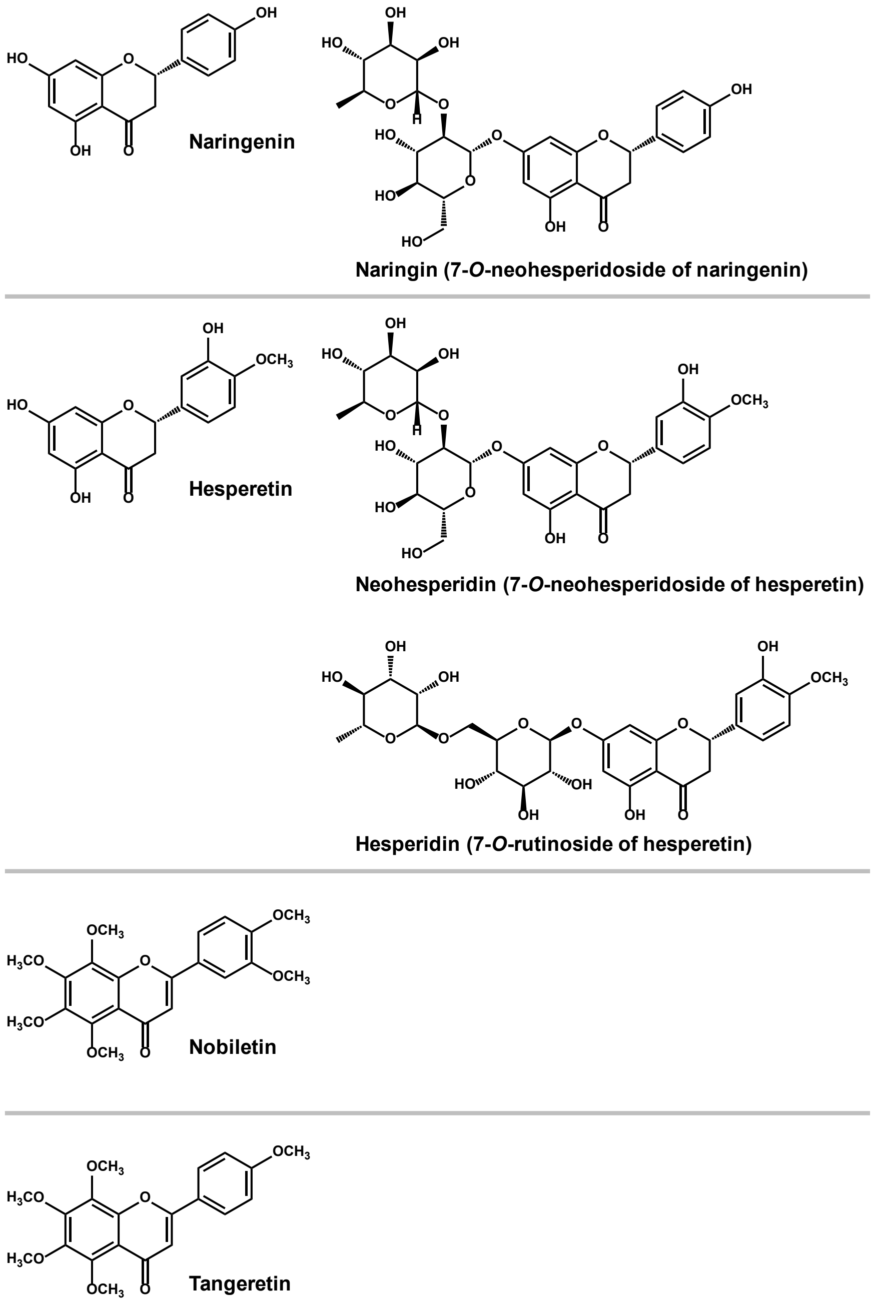

Supplementary Figure 1 Chemical structures of seven *Citrus* flavonoids evaluated in the present study. According to a previous study,[2] the values of the half maximal inhibitory concentration against xanthine oxidase of each *Citrus* flavonoid are 16.5 μM (hesperetin), 107.5 μM (nobiletin), about 200 μM (naringenin) and >200 μM (naringin, neohesperidin, hesperidin, and tangeretin), respectively.

Supplementary Figure 2

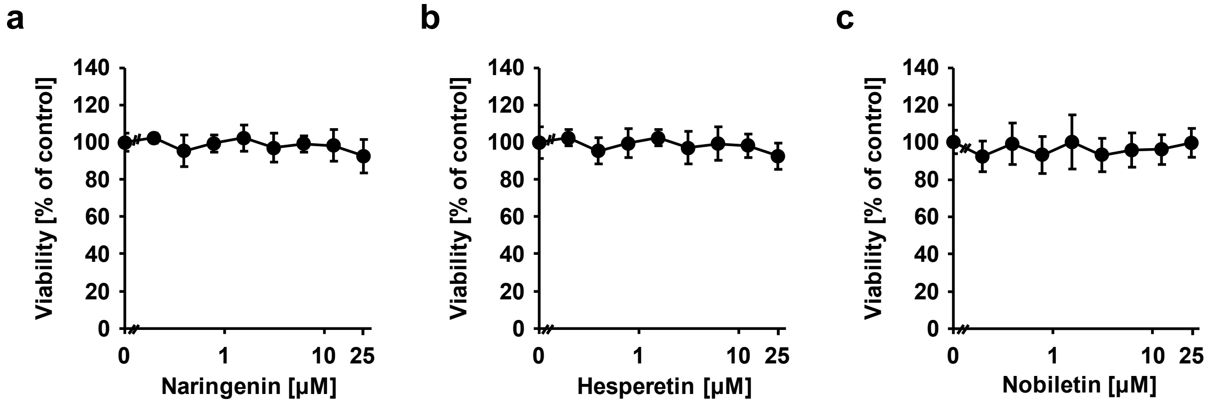

Supplementary Figure 2 Effect of *Citrus* flavonoids on the viability of 293A cells in long term culture. 293A cells were incubated with each flavonoid at indicated concentrations for 48 h. The cell viability (*vs.* vehicle control) was measured by WST-8 assay. Under these experimental conditions, all tested flavonoids had little effect on cytotoxicity, suggesting that these flavonoids could be safe for 293A cells at concentrations of under 25 µM (maximum concentration in this assay). Data are expressed as mean ± SD. $n = 8$.

Supplementary Figure 3

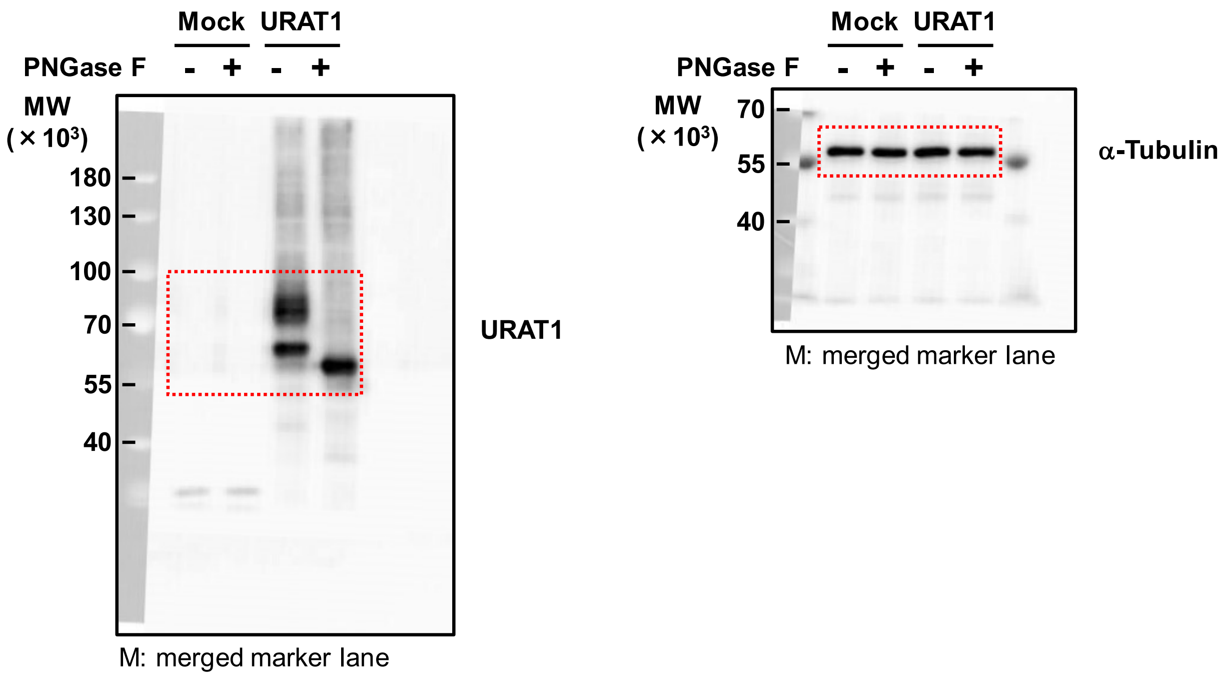

Supplementary Figure 3. Uncropped images of Figure 1a.

Full images of immunoblotting of EGFP-tagged URAT1 (*left*) and α-tubulin (*right*: membrane was sliced before detection based on the molecular size of interested proteins). Blots in each cropped area indicated by red dashed box derived from the same experiment.

Supplementary References

1. Miyata, H. *et al.* Identification of Febuxostat as a New Strong ABCG2 Inhibitor: Potential Applications and Risks in Clinical Situations. *Front. Pharmacol.* **7**, 518 (2016).
2. Liu, K. *et al.* Chemical Evidence for Potent Xanthine Oxidase Inhibitory Activity of Ethyl Acetate Extract of Citrus aurantium L. Dried Immature Fruits. *Molecules* **21**, 302 (2016).
